# Endovascular Treatment of Traumatic Vascular Injuries in the Head and Neck Region

**DOI:** 10.3390/medicina60020269

**Published:** 2024-02-03

**Authors:** Dong Hyun Koh, Ho Cheol Choi, Hwa Seon Shin, Hye Jin Baek, Eun Ha Koh, Mi Jung Park, Dae Seob Choi

**Affiliations:** 1 Department of Medicine, Gyeongsang National University College of Medicine, 816-15 Jinju-daero, Jinju 52727, Republic of Korea; dhgo8559@gmail.com (D.H.K.); sartre81@gmail.com (H.J.B.); ehkohmd@gnu.ac.kr (E.H.K.); 2 Department of Radiology, Gyeongsang National University Hospital, 79 Gangnam-ro, Jinju 52727, Republic of Korea; jaro2@hanmail.net (H.C.C.); ghktjs0315@hanmail.net (H.S.S.); 3 Department of Radiology, Gyeongsang National University Changwon Hospital, 11 Samjeongja-ro, Seongsan-gu, Changwon 51472, Republic of Korea; 4 Gyeongsang Institute of Medical Science, Gyeongsang National University College of Medicine, 816-15 Jinju-daero, Jinju 52727, Republic of Korea

**Keywords:** traumatic vascular injury, carotid artery, vertebral artery, endovascular treatment, stenting, coil embolization

## Abstract

*Background and Objectives*: Traumatic vascular injuries of the head and neck pose significant treatment challenges due to the complex anatomy, diverse clinical presentation, and mostly emergent nature. Endovascular treatment increasingly complements traditional surgical approaches. This study aimed to report our 10-year experience in treating traumatic vascular injuries of the head and neck with endovascular therapy and to determine the effectiveness of endovascular treatment. *Materials and Methods*: A retrospective analysis of 21 patients treated for head and neck vascular injuries between May 2011 and April 2021 was performed. Patients’ medical histories, clinical presentations, imaging findings, treatment materials, and clinical outcomes were reviewed. Treatments included stenting, coil embolization, and other endovascular techniques focused on hemostasis and preservation of the parent vessel. *Results*: The most common injuries involved the internal maxillary artery branches (*n* = 11), followed by the common or internal carotid artery (*n* = 6), vertebral artery (*n* = 3), and others. Endovascular treatment achieved successful hemostasis in all but one case. In five of six carotid artery injuries and two of three vertebral artery injuries, we achieved successful hemostasis while preserving the parent vessel using covered and bare stents, respectively. *Conclusions*: Endovascular therapy might be a useful treatment modality for traumatic vascular injuries in the head and neck region, offering efficacy, safety, and a minimally invasive approach.

## 1. Introduction

Traumatic vascular injuries in the head and neck region pose a significant clinical challenge due to the anatomical complexity and the critical functions of the structures involved. These injuries often lead to high rates of morbidity and mortality, primarily due to risks such as cerebral infarction, hypoxic brain injury, and the inherent challenges in achieving effective hemostasis. The complex anatomy of this region, characterized by a dense network of vital vessels and nerves, further exacerbates the difficulties in diagnosis and treatment. The complexity is heightened by the network of arteries in the head and neck, including the carotid and vertebral arteries, which are critical for blood supply to the brain. The unique anatomy of these vessels and their proximity to other vital structures, such as the spinal cord, cranial nerves, and major airways, add layers of complication to the clinical management and surgical intervention of vascular injury in the head and neck. The causes of such traumatic injuries range from blunt to penetrating trauma, each of which can have devastating consequences if not treated promptly and effectively. This multifaceted challenge underscores the need for meticulous and innovative treatment approaches to manage these critical injuries [1,2].

Historically, the standard treatment for traumatic vascular injury in the head and neck has been surgery, which often carries significant risks and prolonged recovery times. However, with technological advances and the introduction of new medical devices, the treatment landscape has changed, leading to the emergence of endovascular therapy as a frequently preferred and increasingly viable alternative. This minimally invasive technique, encompassing methods like stenting and coil embolization, offers several advantages over traditional surgery. These include less procedural invasiveness, shorter recovery times, and the potential for better patient outcomes. Particularly beneficial in the head and neck region, where surgical access is challenging and open surgery carries a high risk of complications, endovascular therapy enables precise targeting of injured vessels, thereby limiting collateral damage to adjacent tissues. The rapid development of endovascular devices and techniques broadens the spectrum of treatable injuries. This progress is particularly promising for patients with complex vascular traumas, who might have previously been considered unsuitable or at high risk for conventional surgery. These advances in endovascular therapy are redefining treatment options and improving care possibilities for those affected by these challenging injuries [3,4,5].

Despite the apparent benefits of endovascular therapy, its application in the treatment of head and neck vascular trauma remains an evolving field. The evolving nature of this therapeutic approach necessitates ongoing research to elucidate its long-term efficacy, delineate the patient demographics that benefit most, and develop standardized protocols for its application across varying types of vascular injuries. Previous investigations into the utility of endovascular therapy for head and neck vascular trauma have provided valuable insights; however, they are often limited by small sample sizes, a focus on specific types of vascular injuries, and a lack of diversity among patient populations. These limitations underscore the need for more comprehensive studies to build a robust evidence base supporting the use of endovascular therapy in this context.

In this study, we report on our decade-long experience treating various traumatic vascular injuries in the head and neck region with endovascular therapy at our institution. The objective is to evaluate the efficacy and outcomes of endovascular therapy for diverse traumatic vascular injuries in the head and neck region, with a focus on broadening the understanding of its effectiveness across different patient populations and injury types.

## 2. Materials and Methods

This retrospective study was approved by our institutional review board (approval no. GNUH 2023-12-008) on 8 December 2023. Informed consent was waived due to the retrospective nature of the study.

### 2.1. Patients

This retrospective study included 21 patients who underwent endovascular treatment for head and neck vascular injuries at our institution between May 2011 and April 2021. All patients had a clear history of trauma. Patients with vascular injury due to tumor invasion or radiation therapy were excluded.

The medical records and radiological findings of the patients were retrospectively analyzed. The documentation of each case included the patient’s medical history, clinical presentation, computed tomography (CT) and digital subtraction angiography (DSA) findings, the materials used for treatment, and the clinical course.

### 2.2. Angiographic Procedures

**Figure 1:**
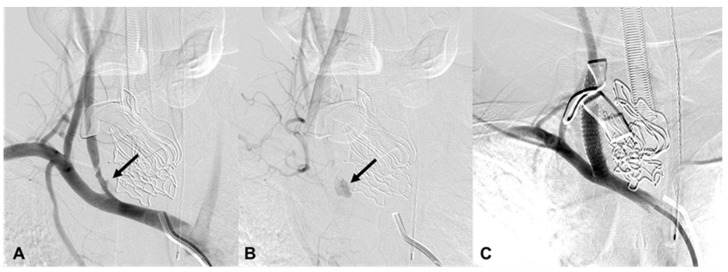
A 32-year-old man (patient 16) with iatrogenic injury in the right common carotid artery (CCA). (**A**,**B**) Brachiocephalic arteriography shows contrast extravasation from the right proximal CCA (arrows). (**C**) After the installation of a covered stent, there is no further contrast extravasation.

Nineteen procedures were performed under local anesthesia, and two were under general anesthesia. The general anesthesia cases were intraoperative iatrogenic vascular injuries of the common and internal carotid arteries (ICA) that failed primary surgical closure and were referred to the angiography suite (Figure 1). All procedures were performed under angiographic control using a biplane or monoplane DSA system (Artis Zee Biplane; Siemens, Erlangen, Germany or Integris Allura; Philips Healthcare, Best, the Netherlands). DSA was used to evaluate the cervical and intracranial vascular anatomy via a percutaneous transfemoral arterial route. The decision to use a stent, coil, particle, adhesive, or a combination of these was made by an interventional neuroradiologist based on the target artery diameter, length, and anatomic location of the lesion.

A 6-French femoral sheath was replaced with a 6- or 8-French shuttle sheath placed in the CCA or vertebral artery (VA) or an 11-French long sheath placed in the aortic arch. The covered or bare stents, coils, particles, glue, or a combination thereof was delivered over the 0.014 or 0.035-inch (330 cm) exchange guidewire or microcatheter and positioned at the level of the bleeding site, including a pseudoaneurysm or extravasation site, using a fluoroscopic roadmap. The investigational devices used in the procedures included the Viabahn self-expanding stent graft (Gore, Flagstaff, AZ, USA), the balloon-expandable Jostent (Abbott Vascular, Redwood City, CA, USA), the SEAL bifurcated stent graft (S&G Biotech, Seongnam, Korea), the Wallstent (Boston Scientific, Malborough, MA, USA), the Enterprise Vascular Reconstruction Device (Codman Neurovascular, Raynham, MA, USA), the LVIS device (MicroVention, Tustin, CA, USA), the Tornado and Nester (Cook Medical, Bloomington, IN, USA), the VortX (Boston Scientific International SA, Natick, MA, USA), the Axium coils (Medtronic, Minneapolis, MN, USA), the Trufill (Codman Neurovascular, Raynham, MA, USA), and the Contour (Boston Scientific International SA, Natick, MA, USA).

During the procedures, heparinized saline was continuously infused through the shuttle or long sheath to prevent in-catheter thrombosis. Systemic heparinization was administered intravenously with a bolus of 3000 IU at the time of stent placement. According to our stenting protocol, triple antithrombotic therapy (heparin, clopidogrel, and aspirin) was recommended after the procedure. Heparin was stopped after 48 h, while clopidogrel was discontinued after six months with a daily dosage of 75 mg. Patients were then left on aspirin alone with a daily dosage of 100–150 mg. Antiplatelet and anticoagulation therapy was administered only when complete hemostasis was determined angiographically and clinically, and not otherwise.

## 3. Results

### 3.1. Causes of Vascular Injury

**Figure 2:**
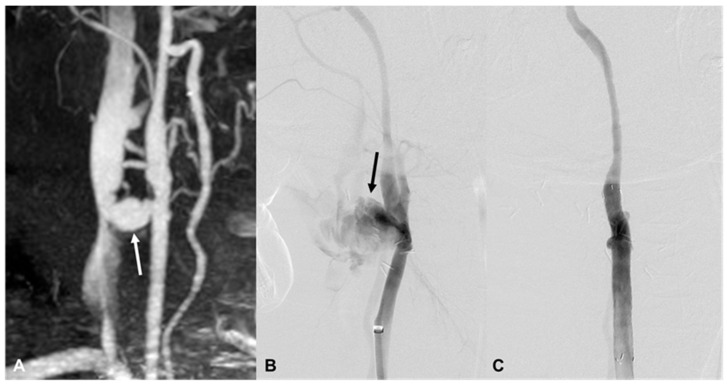
A 47-year-old man (patient 12) with a stab injury. (**A**) CT angiography shows a fistula between the right distal common carotid artery (CCA) and the internal jugular vein (arrow). (**B**) DSA shows massive contrast extravasation from the right distal CCA (arrow). (**C**) The lesion has disappeared after placement of the covered stent.

**Figure 3:**
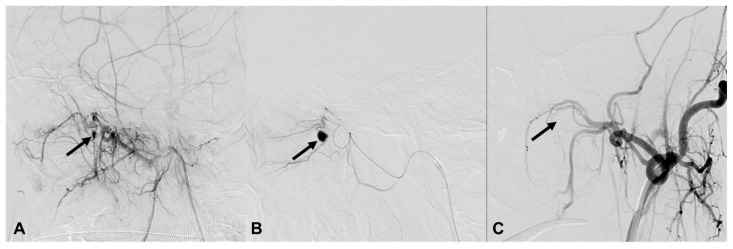
A 23-year-old man (patient 21) with iatrogenic injury in the sphenopalatine branch of the left internal maxillary artery (IMA). (**A**,**B**) Lateral projection of the left IMA angiography shows contrast extravasation from the sphenopalatine branch of the IMA (arrows). (**C**) There is no further contrast extravasation after coil embolization with a 3/2 mm Tornado micro coil (arrow).

Table 1 summarizes the patient characteristics. Of the 21 patients, 15 were male and 6 were female, with a mean age of 48.3 years (range, 22–80 years). All patients had a history of extrinsic trauma or head and neck surgery and presented with massive oral bleeding, epistaxis, neck swelling, or cardiac arrest. Five patients (23.8%) had vascular injuries caused by falls at work or while getting out of bed. Four patients (19.0%) were victims of pedestrian traffic accidents, two (9.5%) had blunt head injuries at construction sites, and four (19.0%) were victims of stab wounds (Figure 1). The remaining six patients (28.6%) had iatrogenic vascular injuries during metastatic lymph dissection of the common carotid artery (CCA) (Figure 2), surgery for hypopharyngeal cancer, two jaw surgeries, nasal septoplasty, or nasal polypectomy (Figure 3).

### 3.2. Injured Vessels

**Figure 4:**
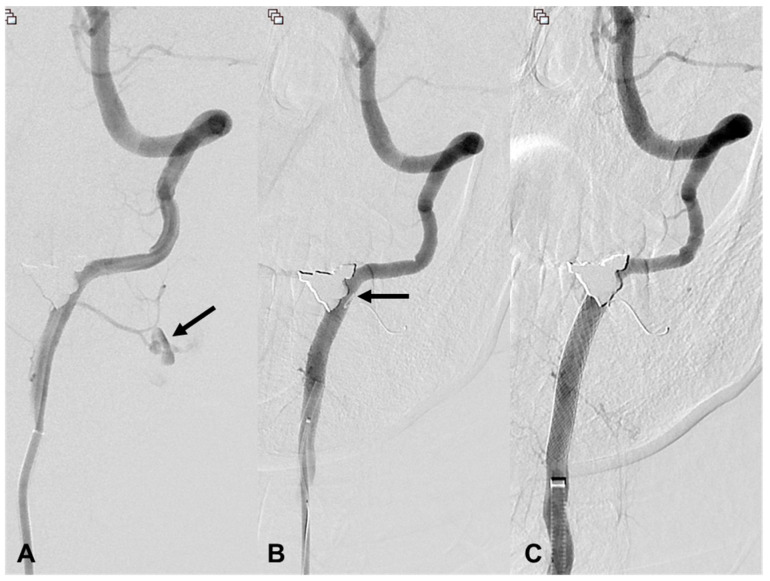
A 53-year-old woman (patient 13) with a stab injury in the left vertebral artery (VA). (**A**) Frontal projection of the left VA angiography shows contrast extravasation from a muscular branch of the left VA V2 segment (arrow). (**B**) After performing coil embolization with a micro coil, no further leakage of contrast is seen; however, a portion of the coil protrudes into the VA lumen (arrow). (C) A stent was inserted to compress the protruding coil against the wall.

**Figure 5:**
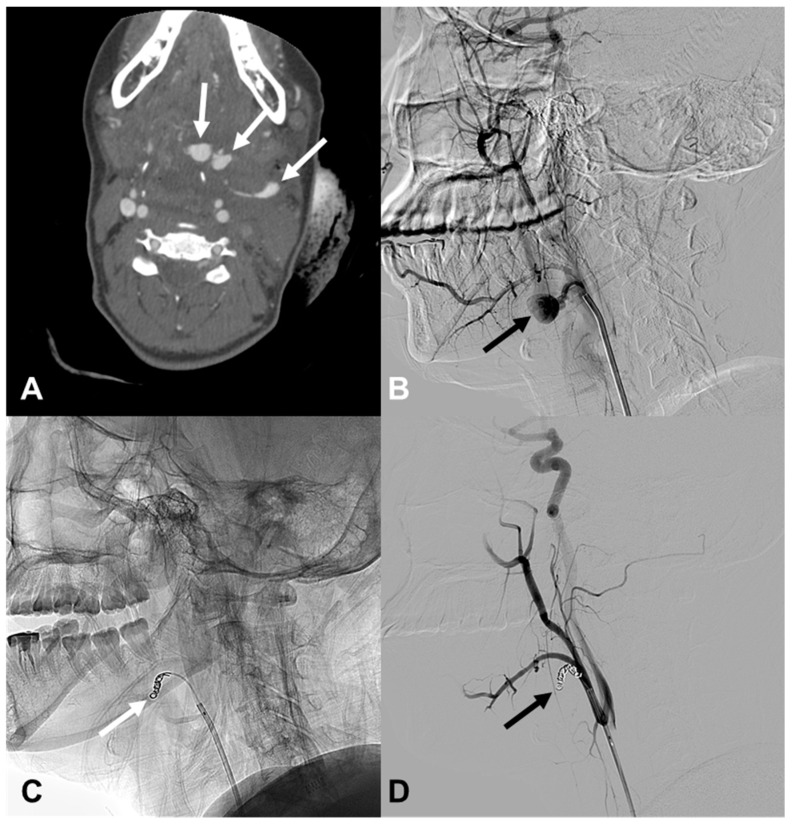
A 22-year-old woman (patient 14) with a stab injury in the left lingual artery. (**A**) Axial CT shows contrast extravasation (arrows) in the left level I and II. (**B**) Left external carotid angiography shows a large pseudoaneurysm at the left lingual artery (arrow). (**C**,**D**) The pseudoaneurysm is no longer opacified after coil embolization with three micro coils (Tornado, 6/2, 5/2, 3/2) (arrows).

The most frequently injured vessels were the internal maxillary artery (IMA) branches (*n* = 11), including the sphenopalatine (Figure 3), middle meningeal, and accessory meningeal arteries, followed by the ICA (*n* = 4), VA (*n* = 3) (Figure 4), CCA (*n* = 2) (Figure 1 and Figure 2), inferior thyroid (*n* = 1), facial (*n* = 1), lingual (*n* = 1) (Figure 5), and ascending pharyngeal (*n* = 1) arteries.

### 3.3. Angiographic Results

Successful hemostasis was achieved with stents or embolic material in all but one patient (patient 1). The patient presented with neck swelling after a fall, and contrast extravasation was seen in the visceral space of the left lower neck on CT. On DSA, contrast extravasation was observed from the left inferior thyroid artery. Embolization was attempted, but the target vessel could not be selected, and embolization could not be performed. Eventually, the patient was transferred to the operating room and underwent ligation of the left inferior thyroid artery.

### 3.4. Carotid Artery Injury

A covered stent was used in all cases of CCA and ICA injuries except for one patient (patient 5). The patient presented with massive epistaxis after a fall-down injury, and CT confirmed multiple facial and sphenoid bone fractures. DSA revealed a carotid-cavernous fistula (CCF) at the right petrous ICA and multiple contrast extravasations at the right IMA branches. Successful embolization of the IMA branches was achieved using two coils and particles (200–250 µm). For the right CCF, balloon-assisted coil embolization was attempted, and ten coils were deployed. However, due to the multiplicity of the CCF and the risk of coil migration into the parent artery, we decided to occlude the ICA with multiple coils after verifying patent collateral blood flow from the anterior and posterior communicating arteries. Post-embolization angiography showed complete occlusion of the ICA and CCF. There were no ischemic events related to the embolization treatment during or after the procedure.

### 3.5. Vertebral Artery Injury

Bare stents were used in three cases of VA injury. In one case, a bare stent was used alone, and in another case, the injured muscular branch of the VA was embolized with a coil and finished with a bare stent (Figure 4). In the third case, up to three overlapping bare stents were deployed, but the bleeding continued, so coils were used to completely occlude the VA.

### 3.6. Complications

No patients died intraoperatively; although, two deaths (patients 10 and 12) occurred during the hospitalization period. In the first case (patient 10), despite complete angiographic hemostasis being achieved with bare stents and micro coils, the patient was declared brain dead 12 days after the procedure due to diffuse traumatic brain injury. In the second case (patient 12), the patient was found in cardiac arrest with a stab wound in the right neck and was brought to the emergency room. After spontaneous circulation was restored, the patient was taken to the operating room for primary repair of the right carotid-jugular fistula identified on CT. However, the primary repair was unsuccessful due to massive bleeding. Hemostasis was achieved with the placement of a SEAL bifurcated stent graft (S&G Biotech, Seongnam, South Korea) (Figure 2), but the patient died of hypovolemic shock 12 h after endovascular treatment. In the other cases, there were no major complications, including thromboembolic events or rebleeding. The follow-up duration ranged from 1 to 2971 days (mean, 593 days).

## 4. Discussion

The management of traumatic vascular injuries in the head and neck region represents a significant challenge in trauma care due to the complex anatomy of the region and the essential functions of the vessels involved. These injuries carry the risk of serious complications such as excessive bleeding, potential stroke, and the possibility of death [1,4]. Our experience in treating 21 patients over a decade underscores the efficacy of endovascular treatment in managing various traumatic vascular injuries in this area. This treatment strategy, which includes the use of stents, coils, and other embolic materials, is consistent with the results of other studies that have demonstrated its efficacy in achieving hemostasis while reducing the risks often associated with traditional open surgical procedures [5,6,7,8].

One of the key findings of our study was the successful use of covered stents in managing carotid artery injuries: five out of the six patients with CCA or ICA injuries were successfully treated with covered stents alone. This finding is supported by other research, which advocates the use of covered stents for their ability to seal arterial disruptions while preserving blood flow, thereby reducing the risk of stroke and other complications [9,10,11,12,13,14,15]. In one patient with CCF, balloon-assisted coil embolization was attempted but failed, and the ICA was sacrificed with multiple coils. What would have been the outcome if a covered stent had been used in this patient? There have been several reports of successful treatment of CCF using covered stents [16,17,18].

In our study, bare stents were used in three cases of VA injury. One was treated with a stent alone, another with a combination of coil embolization and stenting, and the third patient was treated with three overlapping stents. However, bleeding persisted, necessitating complete occlusion of the VA with coils. These procedures resulted in successful hemostasis without complications. This is consistent with the results of Lee et al., who performed endovascular treatment, including stenting and coil embolization in six patients with VA injuries [19]. In their study, except for one patient who underwent coil embolization of a transected VA, all other dissections and pseudoaneurysms were effectively treated with stent placement or stent-assisted coiling with preservation of the parent arteries. There were no delayed neurological or vascular complications or recurrences during a mean follow-up of 36.7 months. This suggests that endovascular treatment is a feasible and safe approach for the treatment of traumatic VA injuries, which supports the results observed in our study. However, it is important to note that long-term follow-up is needed to fully evaluate the safety and efficacy of these treatments. As in our study, there may be cases where stenting with single or multiple stents or with coil embolization does not achieve hemostasis, and VA is sacrificed. In this case, the use of a covered stent may be an option, and several studies have been published recently [15,20,21].

Our study also highlights the limitations of endovascular therapy. For example, the technical difficulty of catheterizing the inferior thyroid artery in one patient underscores the need for continued technical advances and operator expertise. In such cases, surgical intervention remains an important backup option.

Although the deaths in our study were not directly related to the endovascular procedure itself, they highlight the serious nature of head and neck vascular injury. These deaths were associated with complications such as traumatic brain injury and hypovolemic shock, highlighting the importance of comprehensive trauma care beyond the immediate endovascular procedure [3,4].

In our cases, triple antithrombotic therapy was administered after stenting: systemic anticoagulation with heparin for 48 h after the procedure. Clopidogrel and aspirin were also prescribed. Anticoagulation and antiplatelet therapy should be used cautiously in trauma patients because they may increase the risk of rebleeding and, in the presence of concomitant trauma elsewhere, the risk of bleeding in that area. However, stent-related thromboembolic events may occur during or after stent implantation, and a balanced management that considers this is required. In our study, antiplatelet and anticoagulation therapy was used only when complete hemostasis was determined angiographically and clinically, and not otherwise. Most of the other trials used dual antiplatelet therapy with clopidogrel and aspirin [14,16,17,18]. One study used systemic anticoagulation therapy with heparin as in our study, but there were no cases of rebleeding or hemorrhagic complications associated with triple antithrombotic therapy [22]. However, the number of studies is insufficient, and further research is needed.

There are several limitations in our study. The retrospective nature of our research introduces inherent limitations, such as the inability to control for confounding factors and the potential for selection bias. Prospective, randomized trials would be more beneficial to provide more definitive evidence of the efficacy and safety of endovascular approaches. Second, our study included only 21 patients treated over ten years. This relatively small sample size limits the statistical power of our findings and their applicability to a broader population of patients with similar injuries. Third, there was a wide variety of injuries and treatment approaches. The diversity of vascular injuries observed and the corresponding tailored treatment approaches, while demonstrating the flexibility of endovascular therapy, adds variability that may affect the consistency of results and comparative analyses. Finally, although follow-up in our study was up to 2971 days, there was significant interindividual variability. Longer-term follow-up is needed to thoroughly evaluate outcomes. Recognizing these limitations is essential for comprehensively understanding our study’s results and guiding future research. Future studies, ideally larger in scale, multi-center, and prospective in design, are needed to address these limitations and further establish the role of endovascular therapy in managing traumatic vascular injuries in the head and neck area.

## 5. Conclusions

In conclusion, our findings support the growing body of evidence that endovascular therapy is a useful treatment modality for traumatic vascular injuries of the head and neck. This approach offers a balance of efficacy, safety, and a minimally invasive intervention that is crucial in managing these potentially life-threatening injuries. As technology advances and more data become available, we expect treatment strategies to become more refined, leading to improved outcomes for patients with these complex injuries.

## Figures and Tables

**Table 1:** Characteristics of 21 patients treated with endovascular therapy for traumatic injury of the head and neck.

PTs	Age/Sex	Cause	Presentation	CT Findings	DSA Findings	Treatment	Clinical Course
1	73/M	Fall	Neck swelling	EV, visceral space	EV, L inferior thyroid	NA	NA
2	80/F	Fall	Neck swelling	PA, R posterior thyroid	PA, R VA (V2)	bStent	Stable 717 d F/U
3	46/M	Fall	Epistaxis and oral bleeding	Le fort II fracture	EV, L IMA branch	Coil	Stable 260 d F/U
4	57/M	Fall	Bleeding	Facial and sphenoid bone fracture	EV, L IMA branch	Particle	Stable 4 d F/U
5	39/M	Fall	Epistaxis	Facial and sphenoid bone fracture	EV, R IMA branch EV, R cavernous ICA (CCF)	Coil, particle	Stable 39 d F/U
6	51/F	TA	Epistaxis and oral bleeding	Le fort I fracture	EV, R petrous ICA EV, R ascending pharyngeal	cStent, coil	Stable 423 d F/U
7	62/M	TA	Epistaxis	Organizing hematoma, L maxillary sinus	EV, L IMA branch	Coil, glue	Death 911 d d/t esophageal cancer
8	80/F	TA	Massive oral bleeding	Le fort II fracture	EV, L IMA branch EV, both facial	Particle, coil	Stable 651 d F/U
9	61/M	TA	Epistaxis	Le fort II fracture	EV, L IMA branch	Coil	Stable 548 d F/U
10	25/M	Blunt	Ear bleeding	Hematoma, retropharyngeal space	EV, L VA (V2)	bStent, coil	Brain death 12 d
11	64/M	Blunt	Epistaxis and oral bleeding	Le fort II fracture	EV, R IMA branch	Coil	Transferred and lost F/U
12	47/M	Stab	Arrest	R CCA-IJV fistula	EV, R distal CCA	cStent	Death 12 h d/t hypovolemic shock
13	53/F	Stab	Bleeding	Hematoma, L neck	EV, L VA (V2)	Coil, bStent	Stable 1416 d F/U
14	22/F	Stab	Bleeding	PA, L lingual	PA, L lingual	Coil	Stable 190 d F/U
15	34/F	Stab	Bleeding	EV, R ICA	EV, R cervical ICA	cStent	Stable 45 d F/U
16	32/M	Iatrogenic	Massive bleeding during surgery	NA	EV, R proximal CCA	cStent	Stable 2344 d F/U
17	26/M	Iatrogenic	Epistaxis	Normal	EV, R IMA branch	Particle	Stable 102 d F/U
18	58/M	Iatrogenic	Oral bleeding	NA	EV, L cervical ICA	cStent	Death 354 d d/t pneumonia
19	48/M	Iatrogenic	Epistaxis	NA	EV, L IMA branch	Particle, coil	Stable 2971 d F/U
20	34/M	Iatrogenic	Epistaxis	NA	EV, L IMA branch	Particle	Stable 26 d F/U
21	23/M	Iatrogenic	Epistaxis	NA	EV, L IMA branch	Coil	Stable 246 d F/U

Note—EV = extravasation, PA = pseudoaneurysm, CCF = carotid cavernous fistula, R = right, L = left, IMA = internal maxillary artery, cStent = covered stent, bStent = bare stent, ICA = internal carotid artery, CCA = common carotid artery, IJV = internal jugular vein, VA = vertebral artery, TA = traffic accident, NA = not applicable, d = days, h = hours, F/U = follow up.

## Data Availability

Data are contained within the article.
